# Endogenous Retinoic Acid Activity in Principal Cells and Intercalated Cells of Mouse Collecting Duct System

**DOI:** 10.1371/journal.pone.0016770

**Published:** 2011-02-04

**Authors:** Yuen Fei Wong, Jeffrey B. Kopp, Catherine Roberts, Peter J. Scambler, Yoshifusa Abe, Alexandra C. Rankin, Neelanjana Dutt, Bruce M. Hendry, Qihe Xu

**Affiliations:** 1 Department of Renal Medicine, King's College London, London, United Kingdom; 2 Kidney Disease Section, National Institute of Diabetes and Digestive and Kidney Diseases, National Institutes of Health, Bethesda, Maryland, United States of America; 3 Molecular Medicine Unit, Institute of Child Health, London, United Kingdom; 4 Department of Histopathology, King's College Hospital, London, United Kingdom; INSERM, France

## Abstract

**Background:**

Retinoic acid is the bioactive derivative of vitamin A, which plays an indispensible role in kidney development by activating retinoic acid receptors. Although the location, concentration and roles of endogenous retinoic acid in post-natal kidneys are poorly defined, there is accumulating evidence linking post-natal vitamin A deficiency to impaired renal concentrating and acidifying capacity associated with increased susceptibility to urolithiasis, renal inflammation and scarring. The aim of this study is to examine the presence and the detailed localization of endogenous retinoic acid activity in neonatal, young and adult mouse kidneys, to establish a fundamental ground for further research into potential target genes, as well as physiological and pathophysiological roles of endogenous retinoic acid in the post-natal kidneys.

**Methodology/Principal Findings:**

*RARE-hsp68-lacZ* transgenic mice were employed as a reporter for endogenous retinoic acid activity that was determined by X-gal assay and immunostaining of the reporter gene product, β-galactosidase. Double immunostaining was performed for β-galactosidase and markers of kidney tubules to localize retinoic acid activity. Distinct pattern of retinoic acid activity was observed in kidneys, which is higher in neonatal and 1- to 3-week-old mice than that in 5- and 8-week-old mice. The activity was present specifically in the principal cells and the intercalated cells of the collecting duct system in all age groups, but was absent from the glomeruli, proximal tubules, thin limbs of Henle's loop and distal tubules.

**Conclusions/Significance:**

Endogenous retinoic acid activity exists in principal cells and intercalated cells of the mouse collecting duct system after birth and persists into adulthood. This observation provides novel insights into potential roles for endogenous retinoic acid beyond nephrogenesis and warrants further studies to investigate target genes and functions of endogenous retinoic acid in the kidney after birth, particularly in the collecting duct system.

## Introduction

Retinoic acid (RA) is a bioactive molecule derived from dietary vitamin A, which plays an essential role in many basic biological processes such as cell proliferation, differentiation and apoptosis [1]. Acting as a ligand, RA binds and activates heterodimers of retinoic acid receptors (RARs) and rexinoid receptors (RXRs), which are ligand-dependent transcription factors that anchor on the retinoic acid response element (RARE) of retinoic acid target genes [2]. Aside from this classical pathway, RA also affects gene expression via other signaling pathways, in the absence or presence of retinoic acid receptors [1].

Retinoic acid, its synthesizing and metabolizing enzymes, its receptors, as well as its target genes have been widely studied, particularly in the field of developmental biology [3]. In the kidney specifically, Wilson and Warkany first reported that rat fetuses with maternal vitamin A deficiency suffered severe kidney malformation [4]. In the late twentieth century, Mendelsohn et al. observed kidney development impairment in compound mutants of RAR and RXR isotypes [5]. Soon after that, it was found that ablation of a key RA synthesizing enzyme RALDH2 (Raldh2^−/−^) also resulted in defected nephrogenesis [6]. Thus, it has been long appreciated that RA is the primary bioactive vitamin A derivative crucial for nephrogenesis, and that impaired renal development during vitamin A and RA deficiency is due to perturbation of the functional RA-RXR/RAR-RARE pathway.

In contrast to the compelling evidence of RA playing a pivotal role in nephrogenesis, its activity in kidneys after birth is poorly understood, despite emerging data suggesting endogenous RA, upon the accomplishment of its role in nephrogenesis, may have additional functions in the post-natal kidney. We and others had reported the presence of endogenous RA in murine kidneys after birth as measured by high performance liquid chromatography (HPLC) [7]–[11], which may be synthesized locally by RA synthesizing enzymes (RALDH1-4) that are expressed in the kidney [11]–[14]. Furthermore, according to the Nuclear Receptor Signaling Atlas (NURSA) database on tissue-specific expression level of nuclear receptors in adult C57BL/6J and 129X1/SvJ mice, the two most commonly used mouse strains, all six isotypes of retinoic acid receptors (RARα/β/γ and RXRα/β/γ) are expressed in the kidney. More importantly, kidney is among the top two organs that have the highest level of RARα, and among the top five that have the highest level of RARβ in the two mouse strains (http://www.nursa.org/10.1621/datasets). In spite of the contemporary presence of endogenous RA, its synthesizing enzymes and its nuclear receptors, direct proof of endogenous RA being transcriptionally active in the kidney after birth is lacking.

To address this issue, we employed a strain of RARE-hsp68-lacZ transgenic mice, a well-established mouse model of a C57BL/6 genetic background, to detect endogenous RA activity [15]. These mice harbor a lacZ reporter gene driven by an hsp68 minimal promoter with three copies of RARE upstream of the minimal promoter, which is activated by endogenous RA in the presence of its receptors and auxiliary factors, leading to RARE-dependent transcription of lacZ [15]. Expression of lacZ reporter gene can then be detected by X-gal assay and immunostaining of the lacZ gene product β-galactosidase (β-gal). In this model, a strong RA activity was first detected in the metanephric kidneys at embryonic day (E) 11.5–E12.5 [15], during which the ureteric buds invade the metanephric mesenchyme. By employing the same reporter mouse model, Rosselot et al. had recently demonstrated an intense RA activity in the ureteric bud lineage, the precursor of collecting ducts, in E12-E14 kidneys [16]. In this study, we extend the above observations by showing the presence of endogenous RA activity in neonatal, young and adult kidneys, and the activity is confined to the principal cells and intercalated cells of the collecting duct system. Our observations suggest RA activity may play specific roles in these two specialized cell types and lay a foundation for further studies on the target genes and functions of retinoic acid in kidneys after birth.

## Results

### Endogenous RA activity observed in whole-mount kidneys but not liver

**Figure 1:**
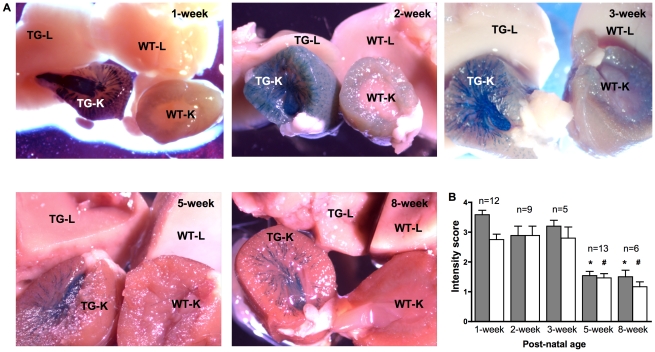
X-gal assay of kidney and liver of *RARE*-*hsp68-lacZ* transgenic (TG) and wild-type (WT) mice. **A**. Fresh tissues were collected and subjected to X-gal assay. Conspicuous X-gal signal (blue) was observed in kidneys of TG mice. Shown here are representative images taken from tissues of 5–13 TG mice and 1–7 WT mice at different time points. Pictures were taken freshly after X-gal assay for 1-, 2-, 5- and 8-week groups; tissues of the 3-week group were fixed in formalin overnight before pictures were taken. TG-K and WT-K: kidney of TG and WT mice respectively; TG-L and WT-L: liver of TG and WT mice respectively. To confirm X-gal signal in the 3-week group, freshly stained tissues were photographed in another instance (Figure S1A). **B**. X-gal signal in kidneys of TG mice was scored semi-quantitatively based on the intensity. 0: no signal; 1: weak signal; 2: moderate signal; 3: strong signal; 4: very strong signal. Gray bars refer to inner medulla of kidney; white bars refer to outer medulla and cortex of kidney. n: number of kidneys from TG mice examined; *: p<0.01 compared to gray bars of 1-, 2- and 3-week old kidneys; #: p<0.01 compared to white bars of 1-, 2- and 3-week old kidneys.

Tissues of wild-type and *RARE-hsp68-lacZ* transgenic mice were examined to differentiate endogenous β-gal, which should be expressed at the same level in both wild-type and transgenic mice, from the specific *lacZ* reporter gene product that should only be observed in transgenic mice. As shown in Figure 1A, kidneys of 1- and 2-week-old wild-type mice demonstrated weak and ubiquitous cortical staining whereas kidneys of 3-, 5- and 8-week-old wild-type mice showed no signal. In contrast, we observed distinct X-gal signal in kidneys of *RARE-hsp68-lacZ* transgenic mice that was equivalent in both male and female at all time points examined. No specific signal was noted in livers of both wild-type and transgenic mice. X-gal signal intensity showed a general decline with age, with kidneys of 1-, 2- and 3-week-old transgenic mice demonstrating a stronger signal compared to kidneys of 5- and 8-week old transgenic mice (Figure 1B). In neonatal kidneys of 1- and 2-day-old mice, a staining pattern similar to that of 1- and 2-week-old mice was observed, whereby X-gal signal was noted in the developing kidneys of the transgenic mice but no signal was observed in the livers of both wild-type and transgenic mice (Figure S1Bi and data not shown).

### RA activity was absent from glomeruli, proximal tubules and thin limbs of Henle's loop

**Figure 2:**
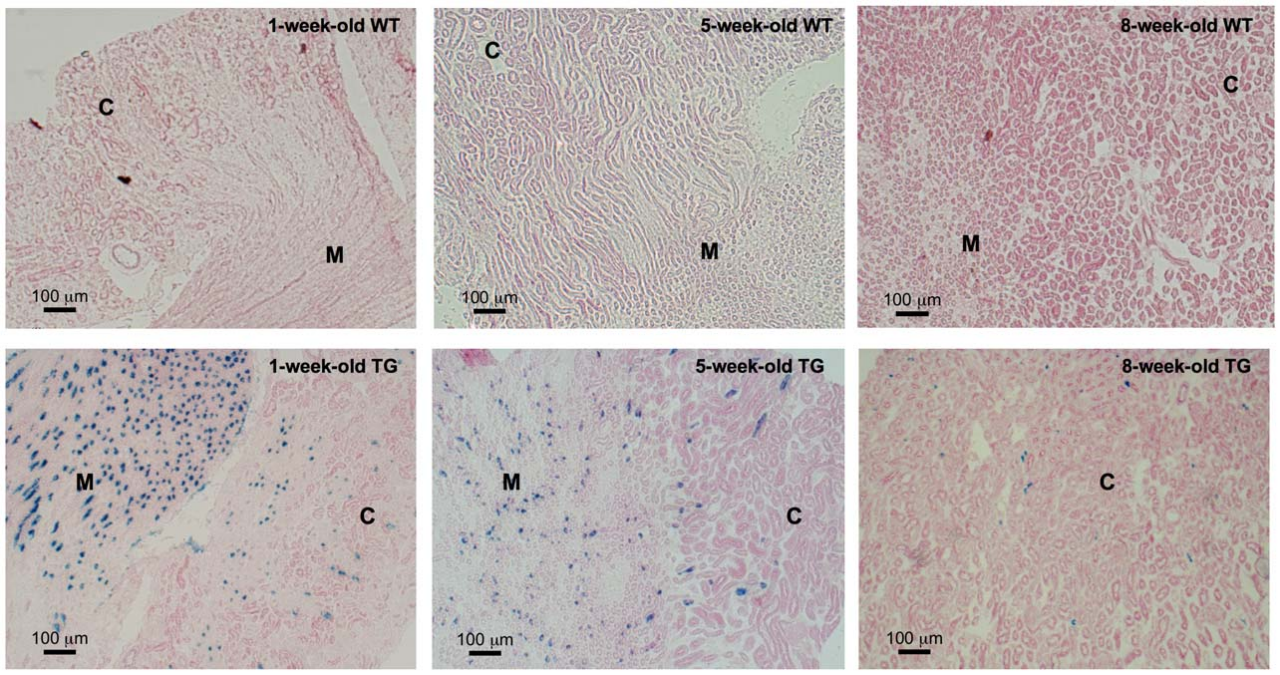
X-gal assay of kidney cryosections. X-gal signal (blue) was detected in kidneys of *RARE-hsp68-lacZ* transgenic (TG) mice but not in wild-type (WT) mice. X-gal signal in the 1-week-old TG mice was more abundant than in the 5- and 8-week-old TG mice. Original magnification was 100×. C: cortex; M: medulla.

**Figure 3:**
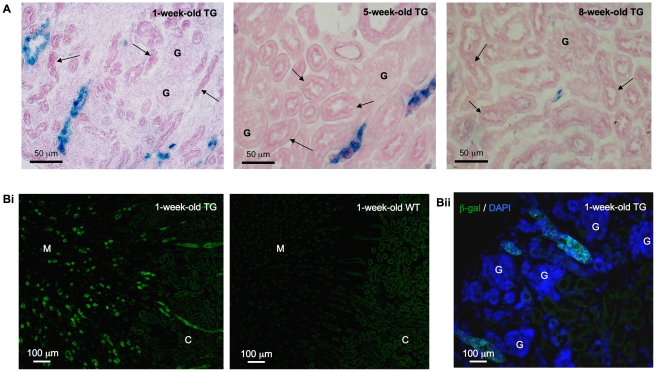
X-gal assay and β-galactosidase (β-gal) immunohistochemistry. **A**. X-gal signal was detected (blue) in a subset of tubules but not in the proximal tubules (arrow) and glomeruli. Original magnification was 400×. **Bi**. β-gal (green) immunostaining in the kidney of *RARE-hsp68-lacZ* transgenic (TG) mice showed a similar staining pattern to X-gal assay (Figure 2) with abundant signal noted in medulla. The background signal that was also noted in the kidney of wild-type (WT) mice might be attributed to endogenous β-gal. **Bii**. Merged image: β-gal signal (green) was observed in tubules and no signal was detected in glomeruli. Nuclei were visualized with DAPI (blue) counterstaining. Original magnification was 100×. No specific signal was noted on sections incubated with non-immune IgG in place of primary antibody (data not shown). C: cortex; M: medulla; G: glomeruli.

In order to confirm our observation of X-gal signal in whole-mount tissues, we performed X-gal assay on cryosections from transgenic and wild-type mice to eliminate the possibility of poor X-gal substrate penetration. In concordance with whole-mount staining, X-gal staining performed on kidney and liver cryosections demonstrated structural specific signal only in kidneys, which was more abundant in the young mice especially in the medulla (Figure 2), but not in livers (Figure S2) of the transgenic mice. Close examination revealed X-gal signal in a subpopulation of tubules, in which some cells exhibited higher staining intensity than others, but no signal was observed in glomeruli and proximal tubules of young and adult mice (Figure 3A). The histological orientation of the X-gal positive tubules containing large and cuboidal cells indicated that those tubules were not the thin limbs of Henle's loop. Since X-gal assay relies on β-gal enzymatic activity that might be affected by tissue fixation [17], the tubular pattern of *lacZ* gene expression and its absence from the glomeruli were confirmed with β-gal immunofluorescence (Figure 3B). In neonatal kidneys of transgenic mice, X-gal signal was observed in the tips of the ureteric bud and the collecting ducts. No signal was observed in the glomeruli, proximal tubules, thin limbs of Henle's loop and the distal tubules (Figure S1Bii).

### Characterization of antibodies as cell type-specific markers

**Figure 4:**
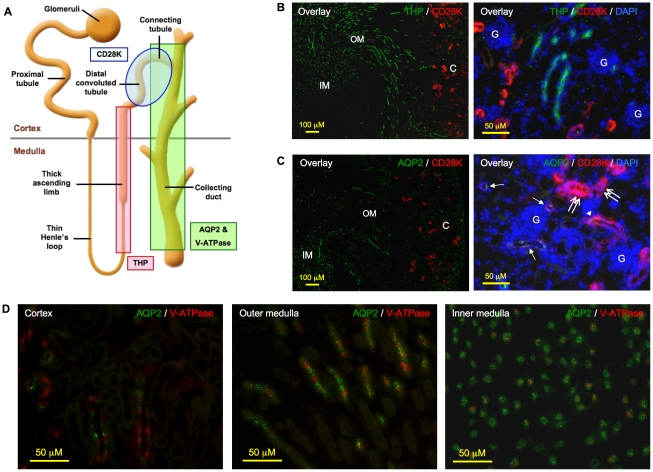
Characterization of antibodies for their specificity in labeling nephron and collecting ducts. **A**. Schematic representation of nephron and collecting ducts. Tamm-Horsfall protein (THP), calbindin D28K (CD28K), aquaporin 2 (AQP2) and vacuolar H^+^-ATPase B1 (V-ATPase) antibodies were used to identify thick ascending limbs, distal convoluted tubules, as well as principal cells and intercalated cells of collecting ducts, respectively. **B**. Left panel (merged image): CD28K signal (red) was observed only in the cortex, whereas THP signal (green) was detected in both cortex and outer medulla, but not in the deep inner medulla. Original magnification was 100×. Right panel (merged image): CD28K (red) and THP (green) signals were mutually exclusive. Original magnification was 400×. **C**. Left panel (merged image): CD28K (red) was detected only in the cortex whereas AQP2 (green) was observed in the outer medulla and inner medulla. Original magnification was 100×; right panel (merged image): distal convoluted tubules stained exclusively and intensely for CD28K (double arrow); tubules stained positive for both CD28K and AQP2 were the connecting tubules (arrowhead) whereas those showing weak or negative CD28K but a positive AQP2 signal were the cortical collecting ducts (arrow). Original magnification was 400×. **D**. Principal cells and intercalated cells that stained positive for AQP2 (green) and V-ATPase (red), respectively, co-localized to the same tubules in the cortex, outer medulla and inner medulla. Original magnification was 400×. No specific signal was noted on sections incubated with non-immune IgGs in place of primary antibodies (data not shown). Shown here are kidney paraffin sections of a 2-week-old mouse. Nuclei were visualized with DAPI (blue) counterstaining. C: cortex; OM: outer medulla; IM: inner medulla; G: glomeruli.

To localize the expression of the *RARE-hsp68-lacZ* reporter gene in the remaining segments of the nephron, a Tamm-Horsfall protein (THP) antibody was used to label the thick ascending limbs [18] while a calbindin D28K (CD28K) antibody was used to identify distal convoluted tubules and connecting tubules, although cortical collecting ducts was also reported to have weaker CD28K expression [18], [19]. To localize the expression of the reporter gene in the collecting duct system that comprises connecting tubules and collecting ducts, AQP2 and V-ATPase antibodies were used to label principal cells and intercalated cells, the two main cell types of the collecting duct system, respectively [18], [20]. We first characterized the specificity of the antibodies by performing double immunofluorescence with different combinations of these antibodies. Results of double immunofluorescence were congruent with their reported histological localization, demonstrating their appropriateness as markers for specific segments of renal tubules and the two cell types in the collecting duct system (Figure 4).

### Lack of RA activity in thick ascending limbs

**Figure 5:**
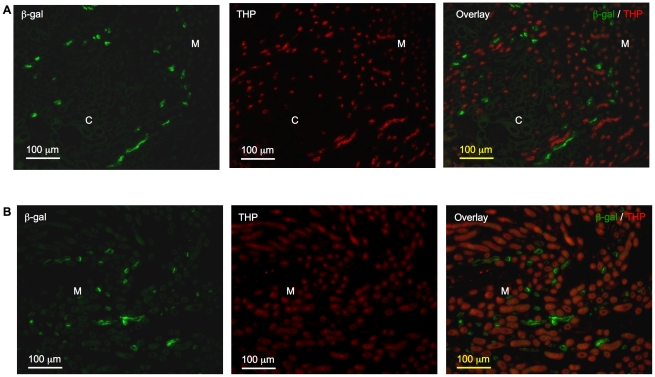
Absence of β-galactosidase (β-gal) signal from the thick ascending limbs. Tubules stained positive for β-gal (green) and Tamm-Horsfall protein (THP, red) were mutually exclusive in kidneys of 2-week-old (**A**) and 8-week-old (**B**) mice. Original magnification was 200×. No specific signal was detected on sections incubated with non-immune IgGs in place of primary antibodies (data not shown). C: cortex; M: medulla.

In kidneys of both young and adult mice, thick ascending limbs, identified by positive THP signal, were distinct from β-gal positive tubules (Figure 5). In kidneys of 1-week-old mice, we observed overlapping THP and β-gal signals in a few individual cells within 2–3 tubules in the kidney cortex (data not shown). Given the rare instances, the biologic relevance of this finding is uncertain.

### RA activity was absent from distal convoluted tubules but likely to be present in connecting tubules and cortical collecting ducts

**Figure 6:**
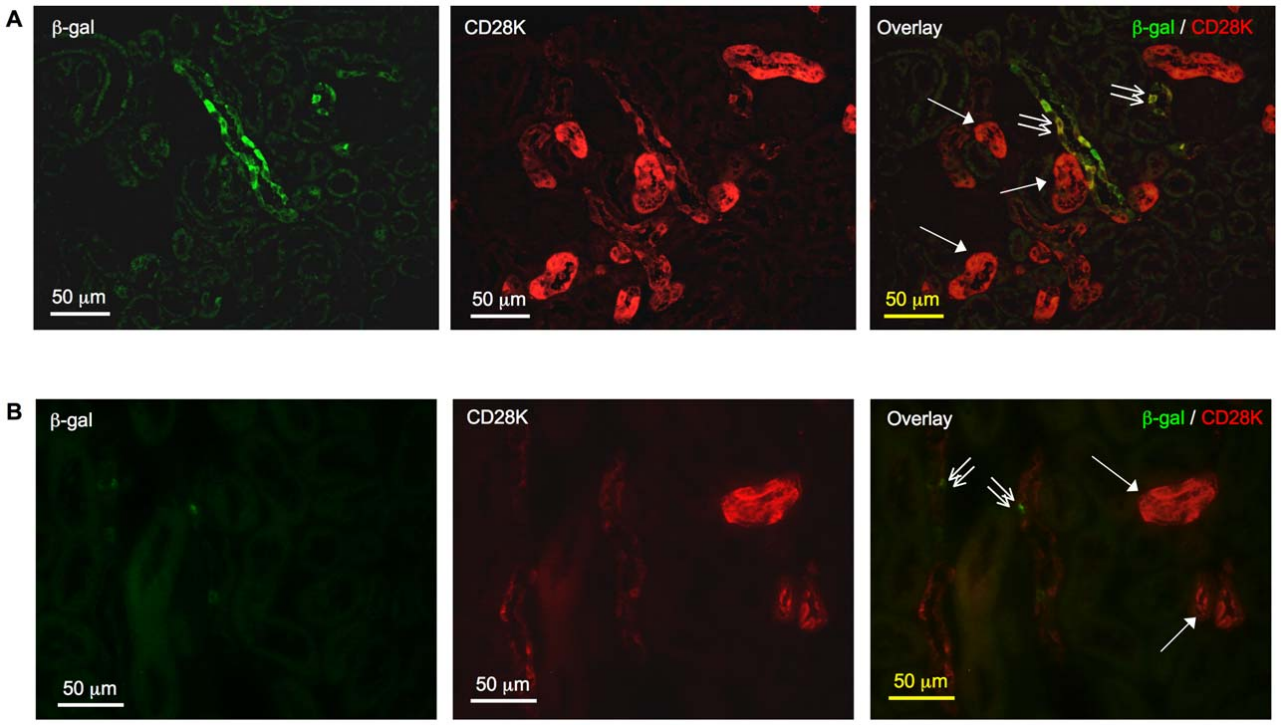
Absence of β-galactosidase (β-gal) signal from the distal convoluted tubules. In kidneys of 2-week-old (**A**) and 8-week-old (**B**) mice, tubules with intense CD28K signal (arrow) did not stain positive for β-gal protein. Some tubules with intermittent CD28K, likely connecting tubules or cortical collecting ducts, demonstrated positive β-gal signal (double arrow). Original magnification was 400×. No specific signal was detected on sections incubated with non-immune IgGs in place of primary antibodies (data not shown).

Tubules with intense CD28K did not demonstrate positive β-gal signal, suggesting the absence of RA activity from distal convoluted tubules. β-gal signal was observed in some tubules with intermittent and relatively weak CD28K signal (Figure 6), which were likely the connecting tubules or cortical collecting ducts, consistent with the presence of CD28K-negative intercalated cells in these segments [19].

### RA activity was observed in principal cells and intercalated cells of collecting duct system

**Figure 7:**
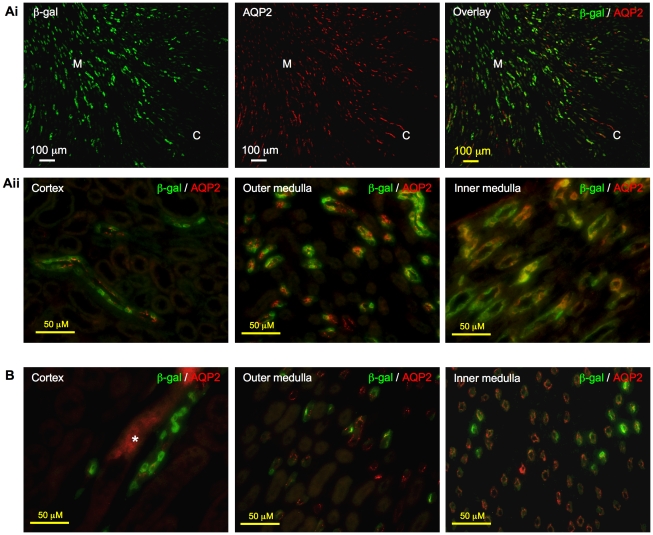
Localization of β-galactosidase (β-gal) to principal cells in collecting ducts. **Ai**. In kidneys of 2-week-old mice, immunostaining pattern of β-gal (green) closely resembled that of aquaporin 2 (AQP2, red). Right panel showed co-localization of the two signals to the same tubules. Original magnification was 100×. **Aii**. Merged images: Principal cells in the cortex, outer medulla and inner medulla appeared yellow-orange when AQP2 signal co-localized with β-gal signal. Original magnification was 400×. **B**. β-gal (green) signal was also observed in principal cells that stained positive for AQP2 (red) in the collecting ducts in cortex, outer medulla and inner medulla of 8-week-old mice. Asterisk indicates non-specific staining, which was also observed on sections incubated with non-immune IgGs in place of primary antibodies (data not shown). Original magnification was 400×.

**Figure 8:**
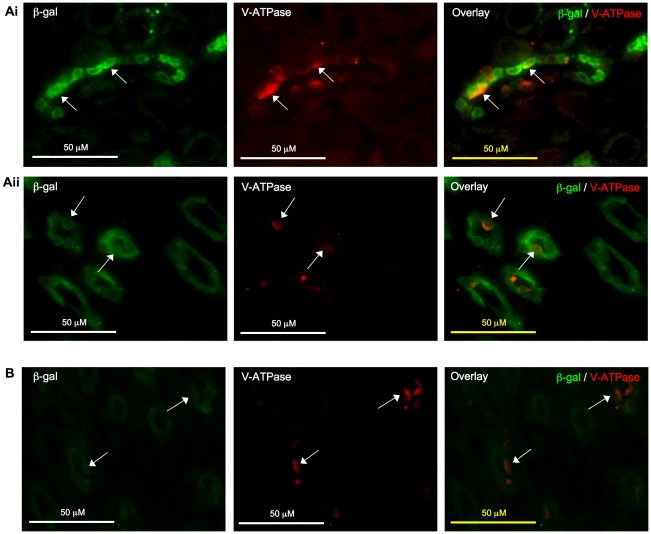
Localization of β-galactosidase (β-gal) to intercalated cells in collecting ducts. β-gal signal (green) was detected in intercalated cells of collecting ducts (arrow) that stained positive for vacuolar H^+^-ATPase B1 (V-ATPase, red) in kidney cortex (**Ai**) and medulla (**Aii**) of 2-week-old mice. In 8-week-old mice, β-gal signal (green) was less apparent in the cortex but was noted in the medulla (arrow) (**B**). Original magnification was 400×. No specific signal was detected on sections incubated with non-immune IgGs in place of primary antibodies (data not shown).

As shown in Figure 7, the immunostaining pattern of β-gal and AQP2 closely resembled each other and the overlay image confirmed the co-localization of the two signals to the collecting duct system. At the cellular level, cells that stained positive for both β-gal and AQP2 were observed in most instances, indicating that the RA activity is most abundant in principal cells, one of the major cell types of the colleting duct system. β-gal expression was more extensive in kidney of young mice, where many cells in the cortex and medulla had dual positive signals (Figure 7A); in older mice, while β-gal expression was less widespread, it was largely localized to principal cells, which was particularly apparent in the medulla (Figure 7B). Although majority of the cells with positive β-gal expression were the principal cells, we found that some intercalated cells, another major cell type of the collecting ducts, also expressed β-gal protein, which was observed in kidneys of both young and adult mice (Figure 8).

## Discussion

The activity of RA is tightly controlled within a coordinated system comprising its synthesizing and metabolizing enzymes to determine its level in the cells [3], its carrier proteins that direct it to different pathways [21], as well as its nuclear receptors and transcriptional co-regulators that confer its transcriptional function [2]. In murine embryonic kidney, the basic components of the retinoid system, i.e. the retinoic acid synthesizing enzymes [6] and the retinoic acid receptors [5], are required to support RA activity during kidney development. Various groups had suggested target genes of RA involved in this regard, including c-ret [22], midkine [23] and LGL1 [24]. In contrast to the well elucidated functions of RA during embryonic nephrogenesis, little is known about its role in kidney after birth. In fact, it remained unknown if RA activity is switched off completely after birth.

If RA activity is of no biological importance in the kidney, from an evolutionary point of view, one would envisage its absence from the organ after birth. However, the existing evidence suggests not only the presence of RA [7]–[11], but also the RA synthesizing enzymes [11]–[14] and the retinoid nuclear receptors (NURSA, http://www.nursa.org/10.1621/datasets). Our observation on the presence of RA activity in the kidney provides evidence that the presence of RA, the RA synthesizing enzymes and the retinoid nuclear receptors is not a mere coincidence, and that the RA-RXR/RAR-RARE pathway that plays an indispensable role during nephrogenesis remains intact and functional in the kidney after birth.

Liver was examined alongside kidney, as it is known to be the major organ for retinoid storage in the body. Endogenous all-*trans* RA, the most abundant isomer of RA, had been reported to be present in the adult murine liver at a level higher than that in the kidney [8]–[10] but our group found that endogenous all-*trans* RA was not measureable in the liver of 3-week-old C57BL/6 mice despite the presence of an abundant all-*trans* vitamin A [7]. The discrepancy may be due to numerous variables including dietary vitamin A content, tissue preparation and retinoid extraction methods, sensitivity of HPLC assays and animal strain or age difference in the homeostasis of endogenous all-*trans* RA. It is a noteworthy observation however that in the *RARE-hsp68-lacZ* transgenic mice, liver was devoid of signal indicating RA activity, in keeping with our HPLC results earlier on the absence of endogenous all-*trans* RA [7]. The very low level of RARs in the liver (NURSA, http://www.nursa.org/10.1621/datasets) may also account for the absence of RA activity.

In this study, we observed that RA activity was present specifically in the ureteric bud-derived cortical and medullary collecting ducts from the neonatal stage up to adulthood, which was reminiscence of the observation from Rosselot *et al.*, where RA activity was noted to be present in both the ureteric bud tip and the ureteric bud trunk in mouse embryonic kidneys at E12-14 [16]. More interestingly, we were able to confirm the presence of RA activity in both major cell types of the collecting ducts, namely the principal cells and the intercalated cells, by employing markers specific to these two cell types. Given that endogenous retinoid nuclear receptor is a prerequisite for the activation of the transgene and that RARβ2 is a direct target gene of RA, we found that some tubules with RA activity indeed expressed RARβ2 protein (Figure S3).

The collecting duct system plays important functions in regulating water transport and acid/base balance, which are roles undertaken by the principal cells and the intercalated cells, respectively. The specific localization of *lacZ* gene expression in the principal cells and the intercalated cells of the collecting duct system indicates that these cells have the appropriate machineries to support transcriptional activity of retinoic acid. It was recently reported that *pkd1* gene, which is expressed in the collecting ducts, is a target gene of RA *in vitro* [25]; a DR1-type RARE was found in 5′-flanking region of *aqp2*, a gene encoding the water channel aquaporin 2 expressed in the principal cells of collecting ducts [26]. We have also noted that post-natal vitamin A deficiency in experimental murine models has been correlated with a series of anomalies including dysregulation of urinary pH and metabolites, some of which are associated with an increased risk of developing urolithiasis [27]–[29], an altered structure and composition of the glomerular and tubular basement membrane [30], and alteration of cytokeratin expression in renal pelvic epithelium [31]. Whether the RA activity in the collecting ducts observed in this study is linked to the specialized functions of principal cells and intercalated cells, and whether it is linked to the regulation of the genes and abnormalities aforementioned, require further investigation.

The ability of RA in stimulating kidney formation has led to postulations that RA may be beneficial in post-natal acquired kidney injury, by modulating its target genes hence re-establishing the developmental program [32], [33]. It has been recently reported in zebrafish, that endogenous RA is required for fin regeneration [34] and for renal progenitor cell expansion [35]. Should the tissue regenerative power of endogenous RA observed in zebrafish be also true in murine kidneys, the localization of endogenous RA activity in the collecting duct system, the only tubular segment that spans across the whole kidney from cortex to the inner medulla, makes it an ideal candidate as first-line repair mechanism to mitigate kidney injury in the collecting ducts as well as other parts of the kidney.

On a different note, an alteration of the endogenous retinoid system in the kidney, including the RA synthesizing enzymes and the retinoic acid receptors, had been reported in various experimental murine models of kidney diseases such as kidney fibrosis [7], diabetic nephropathy [11], glomerulonephritis [36] and puromycin aminonucleoside-induced nephrosis [37], some of which accompanied with a reduction of renal endogenous RA level [7], [11], [36]. It is tempting to speculate that the depletion of endogenous RA from the kidney can be replenished by exogenous RA and hence halt disease progression. However, our recent data failed to demonstrate the therapeutic value of exogenous RA in a transgenic mouse model of kidney fibrosis characterized by a total wipe-out of endogenous RA in the fibrotic kidneys, and when administered at high dose, RA worsened kidney fibrosis [7]. While RA had been identified as one of the promising therapeutic agents in treating various kidney diseases, it is imperative to reconsider how well RA administered exogenously mimics the endogenous RA activity, and whether non-specific activation of the retinoid system in other parts other than the collecting ducts would contribute towards the adverse effects of retinoids.

Early studies are largely devoted to examining the target genes and roles of RA activity in embryonic nephrogenesis. Our data on the presence of RA activity in the kidney collecting ducts support the notion that the retinoid system remains intact and functional after birth, and warrants further studies to unravel the target genes and roles of endogenous RA in the kidney after birth, particularly in the collecting duct system.

## Methods

### Ethics statement

The *RARE-hsp68-lacZ* transgenic mice, which were bred onto a C57BL/6 background, and the related control wild-type mice were fed a standard chow and maintained at the animal facilities at Biological Services, University College London under the approval of the UK Home Office project license PPL 70/6875. All studies on these mice were carried out according to the Animals (Scientific procedures) Act 1986, UK.

### Tissue preparation for histological studies

For cryosections, freshly harvested tissues were submerged in 30% sucrose overnight at 4°C, then transferred to 30% sucrose and Optimum Cutting Temperature (OCT) compound (VWR International Ltd, Lutterworth, UK) at a ratio of 1∶1 before being embedded in OCT and stored at −80°C. Tissues were sectioned at 5 µm and mounted on poly-lysine coated slides (VWR International Ltd). For paraffin sections, tissues were fixed with 10% neutral buffered formalin (pH 7.4) for 16–24 h and were sectioned at 4 µm. For routine histology examination, kidney and liver paraffin sections were subjected to periodic acid-Schiff (PAS) and hematoxylin and eosin staining, respectively.

### X-gal histochemistry

For whole-mount staining, fresh kidneys and livers were harvested from 5-13 *RARE-hsp68-lacZ* transgenic mice and 1–7 wild-type mice. Kidneys with capsule removed, and livers were then cut transversely and fixed for 10 min with phosphate buffered saline (PBS) containing 2% paraformaldehyde and 2 mM MgCl_2_ (pH 7.4) then rinsed with 2 mM MgCl_2_ in PBS twice before being transferred into X-gal staining solution containing 20 mM K_3_Fe(CN)_6_, 20 mM K_4_Fe(CN)_6_.3H_2_O, 2 mM MgCl_2_, 0.01% sodium deoxycholate, 0.02% tergitol NP-40 and 1 mg/ml X-gal substrate (Promega UK Ltd, Southampton, UK) in PBS (pH 7.4), and incubated for 2–48 h at 37°C till desired staining developed. The most representative tissues were selected for photomicrography. For microscopic examination on β-gal localization, OCT embedded tissues from 1- to 8-week-old mice were first sectioned then post-fixed in ice-cold 10% formalin in PBS (pH 7.4) for 10 min. After washing with 3 changes of PBS, 5 min each wash, the cryosections were stained at 37°C with X-gal staining solution containing 5 mM K_3_Fe(CN)_6_, 5 mM K_4_Fe(CN)_6_.3H_2_O, 2 mM MgCl_2_ and 1 mg/ml X-gal substrate in PBS (pH 7.4) for 7 h and 25 h for kidneys and livers, respectively, and then washed with 2 additional changes of PBS. Kidney and liver cryosections were then counterstained with PAS and nuclear fast red respectively, before being coverslipped with Glycergel® (Dako UK Ltd, Ely, UK). For microscopic examination on β-gal localization of 1- and 2-day-old tissues, X-gal stained whole-mount tissues were fixed in zinc formalin (pH 7.4) for 16 h and embedded in paraffin wax, then sectioned at 4 µm and counterstained with PAS and hematoxylin.

### Immunofluorescence

Immunostaining for β-gal was performed on kidney paraffin sections. Sections were dewaxed in 3 changes of xylene, 2 min each, and rehydrated in graded alcohol. Antigen retrieval was performed by pressure cooking slides with 0.01 M citrate buffer at pH 6 for 3 min at full pressure. Sections were then cooled to room temperature and incubated with 0.1 M glycine in PBS for 20 min at room temperature to reduce fixative induced autofluorescence followed by PBS washing for 5 min. Sections were next incubated with 1% bovine serum albumin (BSA) for 2 h at room temperature before incubation with primary antibodies for 1 h at room temperature. The primary antibodies used were: chicken anti-β-gal (dilution 1∶200; Abcam Inc., Cambridge, UK), rabbit anti-aquaporin 2 (AQP2; dilution 1∶200; Milipore UK Ltd, Watford, UK), rabbit anti-Tamm-Horsfall protein (THP; dilution 1∶100; Insight Biotechnology Ltd, Wembley, UK), goat anti- vacuolar H^+^-ATPase B1 (V-ATPase; dilution 1∶500; Insight Biotechnology Ltd), goat anti-calbindin D28K (CD28K; dilution 1∶200; Insight Biotechnology Ltd) and rabbit anti-RARβ2 (dilution 1∶200; Insight Biotechnology Ltd). Non-immune goat, rabbit and chicken IgGs (Insight Biotechnology Ltd) served as negative controls for specificity of primary antibodies. Sections were then incubated with appropriate secondary antibodies (dilution 1∶1000) conjugated with Alexa Fluor 488, Alexa Fluor 555 or Alexa Fluor 568 (Invitrogen Ltd, Paisley, UK). Cell nuclei were counterstained with 4′,6-diamidino-2-phenylindole (DAPI) whenever necessary, before being coverslipped with ProLong® Gold (Invitrogen Ltd). Double immunofluorescence was performed sequentially with overnight 1% BSA incubation at 4°C or 2 h at room temperature before each primary antibody incubation.

### Microscopy

Histological examination was performed on a Nikon Eclipse TE2000-S epifluorescence microscope equipped with a standard RGB filter wheel (Nikon Instruments Europe B.V., Amstelveen, The Netherlands). Images were captured with a DXM1200F Nikon digital camera (Nikon UK Limited, Surrey, UK), and processed and merged with Adobe Photoshop (Adobe Systems Europe Ltd, Uxbridge, UK).

### Statistics

Results are shown as Mean±SE. Statistical significance among multiple groups was evaluated with one-way analysis of variance and p<0.05 was taken as a statistically significant difference.

## Supporting Information

Figure S1 **X-gal assay on kidney and liver of 3-week-old and neonatal** ***RARE-hsp68-lacZ*** **transgenic and wild-type mice.** **A**. Photograph of freshly stained 3-week-old tissues revealed no endogenous X-gal signal in the kidneys of wild-type mice. TG-K and WT-K: kidney of transgenic and wild-type mice respectively; TG-L: liver of transgenic mice. Liver of wild-type mice did not show any signal (data not shown). **Bi**. X-gal signal (blue) was not detected in livers of 2-day-old transgenic (TG) and wild-type (WT) mice. In kidneys of WT mice, endogenous X-gal signal was localized to the inner cortex/outer medulla region while kidney of TG mice showed a distinct staining pattern. TG-K and WT-K: kidney of transgenic and wild-type mice respectively; TG-L and WT-L: liver of transgenic and wild-type mice respectively. **Bii**. Left panel: In kidneys of 2-day-old transgenic mice, X-gal signal (blue) was observed in the ureteric bud-derived collecting ducts and the tips of ureteric bud (arrow). Original magnification was 100 x. Middle and right panels: X-gal signal (blue) was localized to the tips of ureteric bud (black arrow) and collecting ducts in the kidney cortex and medulla. No X-gal signal was observed in the proximal tubules (blue arrow) and glomeruli (red arrow). Original magnification was 400 x.
(TIF)

Figure S2 **X-gal assay on liver cryosections of** ***RARE-hsp68-lacZ*** **transgenic (TG) and wild-type (WT) mice.** No structural specific or cell-specific X-gal signal was detected in livers of both TG and WT mice in all age groups. Shown are liver sections from 1-, 5- and 8-week-old mice. Original magnification was 100 x.
(TIF)

Figure S3 **Immunohistochemistry of β-galactosidase (β-gal) and RARβ2.** In 1-week-old kidney, β-gal signal was observed in tubules that stained positive for RARβ2 receptors (arrow). Original magnification was 400 x. No specific signal was detected on sections incubated with non-immune IgGs in place of primary antibodies (data not shown).
(TIF)
