# Supplementary figures and images for: Endogenous Retinoic Acid Activity in Principal Cells and Intercalated Cells of Mouse Collecting Duct System
Source: PLoS One. 2011 Feb 4;6(2):e16770. doi: 10.1371/journal.pone.0016770 (PMC3033902; doi:10.1371/journal.pone.0016770)

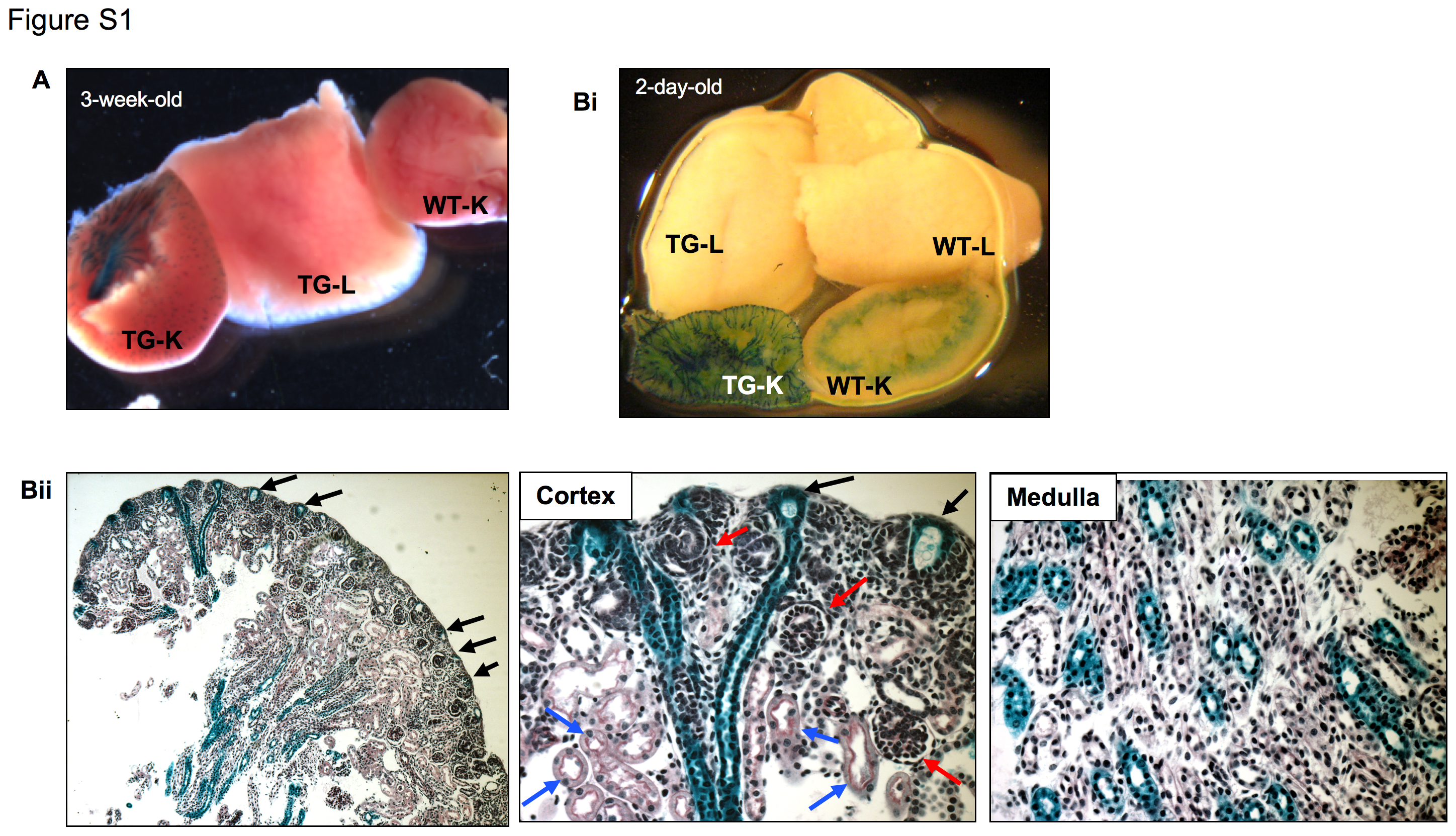

Supplement: Figure S1 — X-gal assay on kidney and liver of 3-week-old and neonatal RARE-hsp68-lacZ transgenic and wild-type mice. A. Photograph of freshly stained 3-week-old tissues revealed no endogenous X-gal signal in the kidneys of wild-type mice. TG-K and WT-K: kidney of transgenic and wild-type mice respectively; TG-L: liver of transgenic mice. Liver of wild-type mice did not show any signal (data not shown). Bi. X-gal signal (blue) was not detected in livers of 2-day-old transgenic (TG) and wild-type (WT) mice. In kidneys of WT mice, endogenous X-gal signal was localized to the inner cortex/outer medulla region while kidney of TG mice showed a distinct staining pattern. TG-K and WT-K: kidney of transgenic and wild-type mice respectively; TG-L and WT-L: liver of transgenic and wild-type mice respectively. Bii. Left panel: In kidneys of 2-day-old transgenic mice, X-gal signal (blue) was observed in the ureteric bud-derived collecting ducts and the tips of ureteric bud (arrow). Original magnification was 100 x. Middle and right panels: X-gal signal (blue) was localized to the tips of ureteric bud (black arrow) and collecting ducts in the kidney cortex and medulla. No X-gal signal was observed in the proximal tubules (blue arrow) and glomeruli (red arrow). Original magnification was 400 x. (TIF) [file pone.0016770.s001.tif]

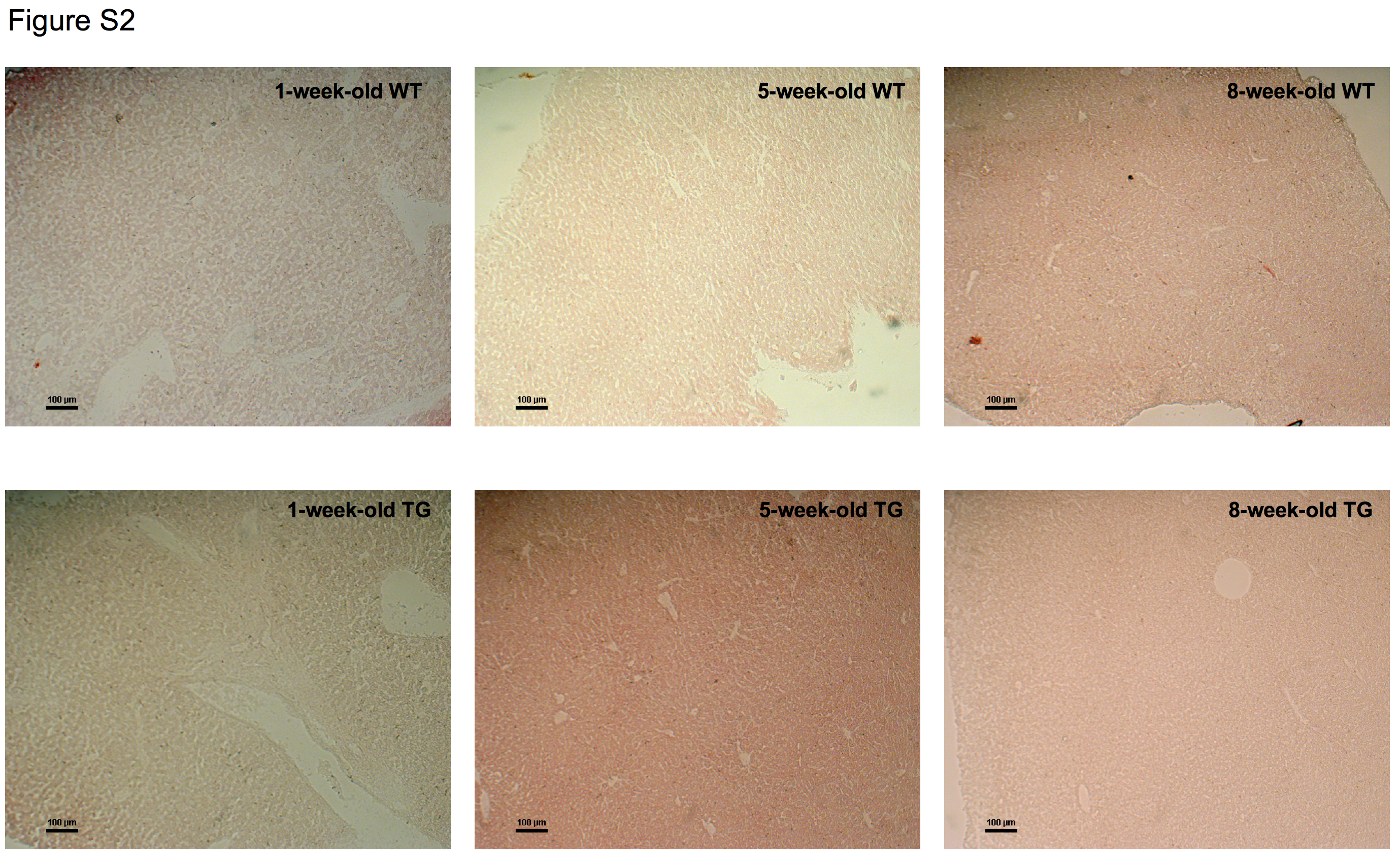

Supplement: Figure S2 — X-gal assay on liver cryosections of RARE-hsp68-lacZ transgenic (TG) and wild-type (WT) mice. No structural specific or cell-specific X-gal signal was detected in livers of both TG and WT mice in all age groups. Shown are liver sections from 1-, 5- and 8-week-old mice. Original magnification was 100 x. (TIF) [file pone.0016770.s002.tif]

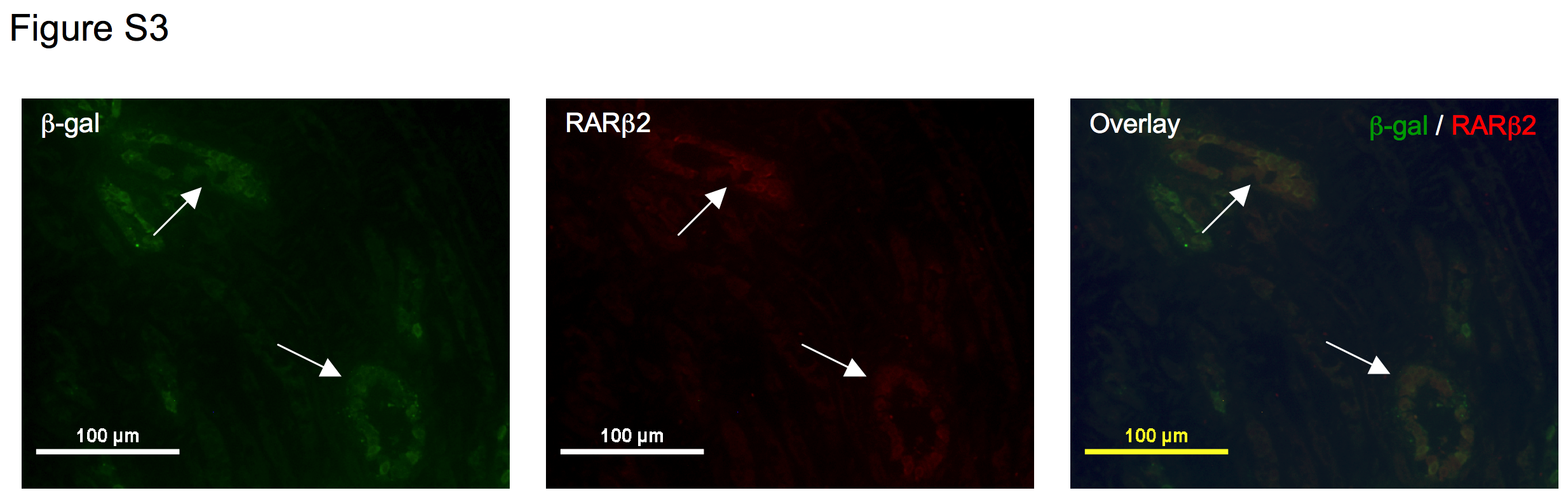

Supplement: Figure S3 — Immunohistochemistry of β-galactosidase (β-gal) and RARβ2. In 1-week-old kidney, β-gal signal was observed in tubules that stained positive for RARβ2 receptors (arrow). Original magnification was 400 x. No specific signal was detected on sections incubated with non-immune IgGs in place of primary antibodies (data not shown). (TIF) [file pone.0016770.s003.tif]
